# Supplementary material for: A specimen of Paralycoptera Chang & Chou 1977 (Teleostei: Osteoglossoidei) from Hong Kong (China) with a potential Late Jurassic age that extends the temporal and geographical range of the genus
Source: PeerJ. 2015 Mar 26;3:e865. doi: 10.7717/peerj.865 (PMC4380157; doi:10.7717/peerj.865)
Supplement: Supplemental Information 1 — Supplementary tables detailing the specimens studied (Table S1) and the phylogenetic characters—and associated codings—used to identify SHGM L275 (Table S2–S6). Chinese language abstracts in Cantonese and Mandarin are also included. [file peerj-03-865-s001.doc]

**Supplementary Information**

**Table S1** Specimens used for comparative studies with SHGM L275. Abbreviations: IVPP, Institute of Vertebrate Paleontology and Paleoanthropology, Beijing, China; WM, National Museums of Tanzania, Dar es Salaam, Tanzania; UALVP, University of Alberta Laboratory for Vertebrate Paleontology, Canada.

| **Specimens studied first hand** | *Paralycoptera* *wui*: IVPP V2989.20, .24, .31, .65, .100, .105, .137  *Sinoglossus lushanensis*: IVPP V6354. 2 |
| --- | --- |
| **Specimens studied in the literature** | *Singida jacksonoides*: WM 490/96, 311/96, 241/96  *Mesoclupea showchangensis*: IVPP V2685.13  *Lycoptera davidi*: IVPP V2328.1, .7, .34  *Yanbiania wangqingica*: IVPP V6767-1  *Tongxinichthys microdus*: IVPP 2332.1  *Hiodon consteniorum*: UALVP 38875  *Huashia gracilis*: IVPP V2996.1  *Jinanichthys longicephalus*: IVPP V10149-51  *Paraclupea chetungensis*: IVPP V816 |

**Table S2A** Phylogenetic matrix showing Shen (1996) characters applicable to SHGM L275 and the codings for it and the most closely-matched taxon. 6 out of 8 applicable characters have states that are the same as *Paralycoptera*.

| **Character number** | **28** | **29** | **30** | **31** | **33** | **34** | **35** | **36** |
| --- | --- | --- | --- | --- | --- | --- | --- | --- |
| SHGM L275 | 1 | 0 | 0 | ? | 2 | 1 | 0 | 2 |
| *Paralycoptera* | 1 | 1 | 0 | 0 | 2 | 1 | 0 | 2 |

**Table S2B** Shen (1996) phylogenetic characters used to identify SHGM L275.

| **Ch #** | **Character description** | |
| --- | --- | --- |
| 28 | The neural spine on the 1st preural centrum: [0] = absent or short; [1] = full neural spine (Taverne 1977, Patterson & Rosen 1977) | |
| 29 | The neural spine on the 1st ural centrum: [0] = absent or short; [1]= full neural spine (Taverne 1977) | |
| 30 | The 1st preural and 1st ural centra: [0] = separate; [1] = fused | |
| 31 | Uroneurals: [0] = three or more pass the 2nd ural centrum; [1] = two or one pass the 2nd ural centrum; [2] = none pass the 2nd ural centrum (Patterson & Rosen 1977, Schultze & Arratia 1988) | |
| 33 | Number of epurals: [0] = three or more; [1] = two; [2] = one; [3] = none (Greenwood 1970) | |
| 34 | Number of hypurals: [0] = seven or more; [1] = six or less (Patterson & Rosen 1977) | |
| 35  36 | Hypurals: [0] = separate; [1] = hypurals of lower lobe fused; [2] = both of lower and upper lobes fused  Number of principal branched rays of caudal fin: [0] = 17 or more; [1] = 16; [2] = fewer than 16 (Patterson & Rosen 1977) | |
| **Table S3A** Phylogenetic matrix showing the Zhang (2006) characters applicable to SHGM L275 and the codings for it and the most closely-matched taxa. 10 out of the 13 applicable characters are coded with the same states as *Paralycoptera*, but for *Singida* 12 are.   | **Character number** | **46** | **47** | **48** | **49** | **50** | **51** | **52** | **53** | **54** | **55** | **56** | **60** | **61** | | --- | --- | --- | --- | --- | --- | --- | --- | --- | --- | --- | --- | --- | --- | | SHGM L275 | 1 | 1 | 0 | 2 | 1 | 0? | 1? | 1 | 0 | 0 | 1 | 0 | 2 | | *Paralycoptera* | 1 | 1 | ? | ? | ? | 0 | 1 | 1 | 0 | 0 | 1 | 0 | 2 | | *Singida* | 1 | 1 | 1 | 2 | 1 | 0 | 1 | 1 | 0 | 0 | 1 | 0 | 2 |   **Table S3B** Zhang (2006) phylogenetic characters used for identifying SHGM L275 (based on the characters of Li & Wilson (1996) and Zhang (1998; 2004)) | |  |
| **Ch #** | **Character description** | |
| 46 | | Length of neural spine on preural 2: [0] = shorter than the neural spine on preural centrum 3; [1] = as long as the neural spine on preural centrum 3 |
| 47 | | Neural spine of preural centrum 1: [0] = rudimentary or short; [1] = long, close to, or reaching the dorsal margin of the body; [2] = absent |
| 48 | | Neural spine on ural centra 1 and 2: [0] = rudimentary; [1] = 1 full; [2] = 2 full; [3] = absent |
| 49 | | Number of epurals: [0] = 3 or more; [1] = 2; [2] = 1; [3] = none |
| 50 | | 1st uroneural reaches: [0] = preural centrum 2 or 3; [1] = preural centrum 1; [2] = ural centra; [3] = no uroneural |
| 51 | | 2 or more uroneurals extending forward beyond the 2nd ural centrum (U3+4): [0] = present; [1] = absent |
| 52 | | Last uroneural much shorter than the first one: [0] = present; [1] = absent; [2] = other condition |
| 53 | | Number of hypurals in adult individuals: [0] = 7 or more; [1] = 6 or less |
| 54 | | Hypural 1 fused but hypural 2 autogenous with ural centra 1 and 2: [0] = absent; [1] = present |
| 55 | | Parhypural of adult individuals fused with preural centrum 1: [0] = absent; [1] = present |
| 56 | | Urodermals: [0] = present; [1] = absent |
| 60  61 | | Anal fin and caudal fin: [0] = separate; [1] = connected  Principal branched caudal fin rays: [0] = 17 or more; [1] = 16 or more; [2] = 15 or fewer; [3] = no separated caudal fin |
| **Table S4A** Phylogenetic matrix showing Wilson & Murray (2008) characters applicable to SHGM L275 and the codings for it and the most closely-matched taxa. 8 out of the 11 applicable characters are coded with the same states as *Paralycoptera* and *Singida*.   | **Character number** | **64** | **65** | **66** | **67** | **68** | **69** | **70** | **71** | **77** | **79** | **80** | | --- | --- | --- | --- | --- | --- | --- | --- | --- | --- | --- | --- | | SHGM L275 | 0 | 2 | ? | 0 | 1 | 0 | 0 | 1 | 0 | 0 | 0 | | *Paralycoptera* | ? | 2 | 0 | 0 | ? | 0 | 0 | 1 | 0 | 0 | 0 | | *Singida* | 0 | 2 | 1 | 1 | 2 | 0 | 0 | 1 | 0 | 0 | 0 |   **Table S4B** Wilson & Murray (2008) phylogenetic characters used for identifying SHGM L275. | |  |
| **Ch #** | **Character description** | |
| 64  65 | | Posterior end of anal fin: [0] = separate from caudal fin; [1] = continuous with caudal fin  Number of principal caudal fin rays: [0] = 19 or more; [1] = 18; [2] = 17 or fewer |
| 66 | | Uroneurals: [0] = 3 or more; [1] = 2 or 1; [2] = absent |
| 67 | | Neural spine on ural centrum 1: [0] = absent or rudimentary; [1] = 1 or more |
| 68 | | Epurals: [0] = 2 or 3; [1] = 1; [2] = absent |
| 69 | | Neural spine on the 1st preural centrum: [0] = complete; [1] = rudimentary; [2] = absent |
| 70 | | Number of neural spines on the 2nd preural centrum: [0] = 1; [1] = 2 |
| 71  77 | | Number of hypurals: [0] = 7; [1] = 6 or fewer  Upper hypurals and second ural: [0] = not fused; [1] = fused |
| 79 | | Dorsal fin shape: [0] = base moderately long, fin triangular or falcate; [1] = base very short, much shorter than fin height, or fin absent; [2] = base moderately long to very long, fin with rounded outline anteriorly and posteriorly |
| 80 | | Posterior rays of dorsal and anal fin: [0] = shorter than anterior ones; [1] = longer than or as long as anterior ones |

**Table S5A** Phylogenetic matrix showing the Xu & Chang (2009) characters applicable to SHGM L275 and the codings for it and the most closely-matched taxon. 6 out of 8 applicable characters have states that are the same as *Paralycoptera* and *Singida*. *This character state should be 1, ‘completely developed’, as the original entry is an error that contradicts the main body of Xu & Chang’s (2009) paper.

| **Character number** | **54** | **55** | **56** | **57** | **59** | **60** | **61** | **62** |
| --- | --- | --- | --- | --- | --- | --- | --- | --- |
| SHGM L275 | 1 | 0 | 1 | 1 | 1 | 0 | 0 | 2 |
| *Paralycoptera* | 0* | 1 | 1 | 1 | 1 | 0 | 0 | 2 |
| *Singida* | 0 | 1 | 1 | 1 | 1 | 0 | 0 | 2 |

**Table S5B** Xu & Chang (2009) phylogenetic characters used for identifying SHGM L275.

| **Ch #** | **Character description** |
| --- | --- |
| 54 | Neural spine on pu1: [0] = rudimentary or absent; [1] = completely developed |
| 55 | Neural spine on u1: [0] = rudimentary or absent; [1] = completely developed |
| 56 | Epural: [0] = two or more; [1] = one or absent |
| 57 | Number of uroneurals: [0] = five or more; [1] = two to four; [2] = one; [3] = absent |
| 59 | Number of upper hypurals: [0] = five or more; [1] = four or fewer |
| 60 | Most upper hypurals: [0] = independent; [1] = fused with last centrum |
| 61  62 | Lower hypurals: [0] = two, separated or partly fused; [1] = one large hypural; [2] = three, anterior two partly joined proximally  Principal branched caudal fin rays: [0] = 17 or more; [1] = 16; [2] = 15 or fewer |

**Table S6** Spore taxa found in the Lai Chi Chong Formation and their temporal ranges (based on data from Lee *et al.* 1997 and fossilworks.org). Taxa in bold font are the most abundant ones in the formation.

| **Taxon** | **Temporal range (Ma)** |
| --- | --- |
| **Ferns**  ***Cicatricosisporites minutaestriatus***  ***Cicatricosisporites subrotundus***  *Pilosisporites verus*  ***Cicatricosisporites australiensis***  ***Cyathidites australis***  *Trilobosporites sp.*  ***Cyathidites minor***  ***Klukisporites pseudoreticulatus***  *Todisporites minor*  *Osmundacidites wellmanii*  *Neoraistrickia sp.*  *Apiculatisporis sp.*  *Punctatisporites*  *Lophotriletes sp.*  *Acanthotriletes sp.* | 112.6 - 109.0  122.46 - 112.6  125.45 - 99.7  136.4 - 66.043  136.4 - 61.7  145.5 - 70.6  164.7 - 5.332  183 - 5.332  196.5 – 5.332  252.3 - 66.043  383.7 - 66.043  409.1 - 99.7  439.0 - 99.7  383.7 - 251.3  409.1 - 66.043 |
| **Gymnosperms**  ***Classopollis classoides***  ***Classopollis annulatus*** | 196.5 - 66.043  205.6 - 61.7 |
| *Abietineaepollenites sp.*  *Podocarpidites sp.*  *Protopinus sp.*  *Pinuspollenites sp.*  *Ginkgocycadophytus nitidus*  *Psophosphaera sp.*  *Ephedripites sp.* | 161.2 - 23.03  196.5 - 5.332  235.0 - 201.6  247.2 - 33.9  259.0 - 55.8  265.0 - 112.6  268.0 - 40.4 |

**Chinese-version abstract**

Below are traditional and simplified Chinese versions of the paper’s abstract to allow non-English-speaking Chinese audiences to read the paper’s main conclusions.

**Abstract** **S1** (Traditional Chinese):

**香港首個中生代魚化石研究**

謝子旗 文嘉棋 張彌曼

摘要：我們在2013年在香港大學地球科學系的收藏中發現一塊從荔枝莊收集的魚化石。透過顯微鏡觀察與文獻比較，該魚鑒定為伍氏副狼鰭魚(*Paralycoptera*)。這項研究擴展了副狼鰭魚的地理範圍，並把副狼鰭魚的年代推前至晚侏羅紀。此外，香港化石可能隱藏著更多的研究問題和答案，故香港化石可成為今後的研究目標。這項研究提供了對瞭解香港中生代脊椎動物群的重要一步。

關鍵字：中生代，魚，香港，副狼鰭魚，荔枝莊組

**Abstract** **S2** (Simplified Chinese):

**香港首个中生代鱼化石研究**

谢子旗 文嘉棋 张弥曼

摘要：我们在2013年在香港大学地球科学系的收藏中发现一块从荔枝庄收集的鱼化石。透过显微镜观察与文献比较，该鱼鉴定为伍氏副狼鳍鱼(*Paralycoptera*)。这项研究扩展了副狼鳍鱼的地理范围，并把副狼鳍鱼的年代推前至晚侏罗纪。此外，香港化石可能隐藏着更多的研究问题和答案，故香港化石可成为今后的研究目标。这项研究提供了对了解香港中生代脊椎动物群的重要一步。

关键词：中生代，鱼，香港，副狼鳍鱼，荔枝庄组

**Supplemental References**

**Patterson C, Rosen DE. 1977.** Review of the ichthyodectiform and other Mesozoic fishes and the theory and practice of classifying fossils. *Bulletin of the American Museum of Natural History* 158:81–172.

**Schultze HP, Arratia G. 1988.** Reevaluation of the caudal skeleton of some actinopterygian

fishes. 11. Hiodon, Elops and Albula. *Journal of Morphology* **195**:257–303

DOI 10.1002/jmor.1051950304.

**Taverne L. 1977.** Osteologie, phylogenese et systematique des teleosteens fossiles et actuels du super-ordre des ost«eoglossomorphes, premiere partie. Osteologie des genres Hiodon, Eohiodon, Lycoptera, Osteoglossum, Scleropages, Heterotis et Arapaima. *Academie Royale de Belgique, Memoires de la Classe des Sciences* **42**:1–235.

**Zhang JY. 1998.** Morphology and phylogenetic relationships of *Kuntalunia*

(Teleostei: Osteoglossomorpha). *Journal of Vertebrate Paleontology* **18(2)**:280–300

DOI 10.1080/02724634.1998.10011057.

**Zhang JY. 2004.** New fossil osteoglossomorph from Ningxia, China. *Journal of Vertebrate*

*Paleontology* **24(3)**:515–524 DOI 10.1671/0272-4634(2004)024[0515:NFOFNC]2.0.CO;2.
